# Annular Microfluidic Meta-Atom Fusion-Enabled Broadband Metamaterial Absorber

**DOI:** 10.1007/s40820-025-02018-2

**Published:** 2026-01-05

**Authors:** Jinpeng Peng, Yi Zhang, Zihao Chen, Qiye Wen, Shaomeng Wang, Yaoyao Li, Aiwu Zhou, Zhengnan Sun, Xiaohui Mu, Xiaosheng Zhang

**Affiliations:** 1 School of Integrated Circuit Science and Engineering, University of Electronic Science and Technology of China, Chengdu, 611731 People’s Republic of China; 2 Tianfu Jiangxi Laboratory, Chengdu, 641419 People’s Republic of China; 3 School of Electronic Science and Engineering, University of Electronic Science and Technology of China, Chengdu, 611731 People’s Republic of China

**Keywords:** Metamaterial absorber, MMA, 3D printing, Additive manufacturing, Radar stealth

## Abstract

Electromagnetic (EM) metamaterial absorbers (MMAs) with broadband absorption are of growing interest for applications such as stealth and EM interference mitigation. In this work, we present a novel 3D-printed MMA based on a fused annular microfluidic meta-atom (FAMMA) architecture, designed for W-band absorption. The FAMMA structure features three kinds of orthogonally fused annual meta-atoms, forming a complex 3D microfluidic meta-atom with intricate architecture. Fabricated via high-precision micro 3D printing technology, the FAMMA-based MMA exploits the synergistic solid–liquid coupling effect of the unique three-dimensional orthogonal structure to achieve strong broadband absorption. Three representative FAMMAs with different geometric dimensions have achieved ultra-low reflection loss (RL of − 42.1 dB), ultra-broadband effective absorption bandwidth (EAB of 31.3 GHz), and dual-band absorption (in 76.0–85.3 and 99.1–105.6 GHz), respectively. The underlying absorption mechanisms are elucidated by impedance matching theory and electromagnetic field distribution analyses. Application demonstrations show that the FAMMA-based MMA significantly suppresses radar echo power and renders metallic targets undetectable to both radar detector and radar imaging systems, highlighting its potential in stealth technology. Overall, this work establishes a new design concept for high-performance broadband millimeter wave MMAs, opening new avenue for future applications such as high-speed communication, through-wall sensing, and drone detection.

**Supplementary Information:**

The online version contains supplementary material available at 10.1007/s40820-025-02018-2.

## Highlights

- Unique 3D-printed electromagnetic metamaterial absorber based on a fused annular microfluidic meta-atom architecture.
- Synergistic solid-liquid coupling effect of the unique three-dimensional orthogonal structure to achieve strong broadband absorption.
- High-performance broadband absorption for millimeter wave.

**Supplementary Information:**

The online version contains supplementary material available at 10.1007/s40820-025-02018-2.

## Graphical abstract

**Figure:**
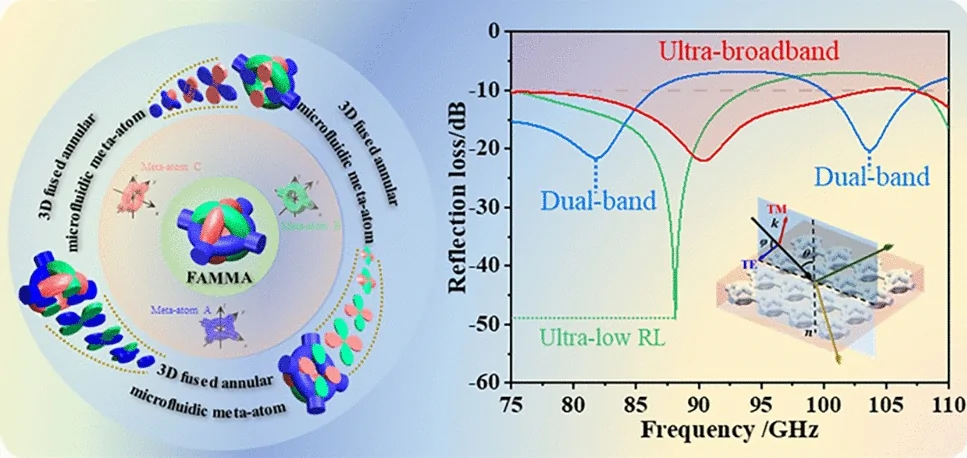

## Introduction

Electromagnetic (EM) metamaterial absorber (MMA) represents a transformative class of artificially engineered materials designed to manipulate EM waves in ways unparalleled by traditional absorbing materials [1–3]. Unlike traditional materials, whose absorption characteristics are governed by their intrinsic material dielectric and magnetic loss, MMAs interact with EM waves via a meta-atom lattice, achieving near-unity absorption through meticulous structural design to maximize EM wave confinement and dissipative loss [4]. Previously reported water-based MMA almost operates in the low-frequency microwave band. It is noteworthy that high-frequency millimeter wave radars have already demonstrated promising potential across a wide range of applications, including high-speed communication [5], automobile driver-assistance systems [6], through-wall sensing [7], and drone detection [8]. For example, short-range wireless communication technology, such as WiGig, leverages the 60 GHz frequency band to facilitate high-speed transmission with a theoretical speed of 7 Gbps. In automotive industry, vehicle-mounted millimeter wave radar systems operating at 79 GHz offer substantially higher angular resolution than traditional microwave radar, which enables accurate identification of pedestrians, vehicles, and small obstacles on the road. It is one of the key sensor technologies for achieving autonomous driving. In addition, the penetrability of millimeter waves is exploited in imaging systems to realize harmless detection and imaging of the human body, in which are already deployed in security and medical settings such as airports, custom checkpoints, and hospitals.

MMAs with broadband absorption are highly promising for a diverse range of applications including wireless communication [9, 10], radar stealth [11], EM interference shielding [12], and optical cloaking [13, 14]. However, the inherent periodic nature of MMAs often leads to resonant absorption confined to narrow bands. To overcome this limitation, strategies, such as structural coupling or gradient geometry [15–18], have been employed to extend the absorption band via resonance superposition. Xu et al. conducted extensive research in kirigami-inspired EM metamaterial for independent control frequency, bandwidth, and amplitude [19]. Furthermore, a strip array of ITO films on novel metapyramid structure based on impedance-gradient theory is reported to achieve wide-angle broadband absorption [20]. And a composite metasurface can asymmetrically control transmitted microwave wavefront under radar-IR bi-stealth scheme [21]. Inspired by atomic doping in photonic materials, Qin et al. exploited mutual coupling between water meta-atoms of different sizes to ensure impedance matching and broaden EM wave absorption [22]. Despite these advances, the on-going pursuit for high-performance broadband MMAs faces two critical challenges. First, while the absorption bandwidth generally scales with the number of resonant structures, the spatial density of these structures is constrained by planar packing limit due to steric hinderance. For instance, small meta-atoms can only be “doped” into the limited interstitial space between large meta-atoms, imposing a practical ceiling on integration density and absorption performance. Second, as architectures of MMAs become increasingly intricate, conventional manufacturing techniques often struggle to realize these sophisticated structures, constrained by material and process compatibility [23, 24]. Consequently, the central challenge in broadband MMA development lies in designing meta-atoms while reliably producing these intricate designs to achieve broadband and strong absorption characteristics.

To address this challenge, in this work, we present a previously unexplored design and fabrication paradigm for broadband MMAs based 3D “meta-atomic fusion” enabled by high-precision 3D-printed microfluidics. We introduce a design strategy of FAMMA, which describes the integration of three planar annular meta-atoms orthogonally into a 3D FAMMA and significantly increases the packing density compared to 2D planar “meta-atomic doping,” enabling synergistic resonant coupling and broadband absorption with inherent polarization insensitivity and adaptability over a large range of incident angle. The use of liquid dielectric core offers exceptional design flexibility, allowing us to arbitrarily shape the resonant structures [25–27]. Importantly, the FAMMA architecture is realized through a 3D microfluidic network fabricated via a high-precision micro 3D printing technology [28], overcoming the fabrication challenges that limited the structural complexity and hence the performance of MMAs. Altogether, FAMMA introduces a fundamentally new approach to MMA design based on meta-atomic fusion, paving the way for the development of high-performance broadband millimeter wave MMA.

## Experimental Section

### Equipment and Materials

The experimental equipment and materials used in this study include a high-resolution PμSL 3D printer (MicroArch S240, Boston Microfabrication BMF), photosensitive resin (HTL, Boston Microfabrication BMF), and the mechanical property parameters of HTL resin are shown in Fig. S1 a terahertz time-domain spectrometer (FicoTM, Zomega), scanning electron microscope (Thermoscientific Apreo 2S and Oxford Ultim Max 40), a vector network analyzer (AV3672C), standard gain horn antenna (Hengda Microwave), a FMCW-based radar (Lytid), a radar detector (Microbrain), and an FDM 3D printer (Bambu Lab X1 Carbon).

### FAMMA Fabrication

The FAMMA-based MMA was designed using SolidWorks, and sliced into 2D patterns for printing by PμSL. The 3D-printed device was placed in an ultrasonic bath filled with anhydrous ethanol to remove residual resin. Compressed air was then applied to clear microchannels. The ultrasonic cleaning and air ventilation steps were repeated to ensure the channel was clear from residual resin.

To fill the FAMMA-based MMA with water, the cleaned and dried device was immersed in water and placed in a vacuum chamber for 2 h. After that, all inlets of the water-filled device were sealed with scotch tape to prevent water leakage. Finally, an aluminum foil was adhered to the bottom surface of the FAMMA device as an anti-transmission layer.

### Absorption Testing Setup

RL measurements of the FAMMA-based MMA were conducted in a microwave anechoic chamber. The testing setup included a vector network analyzer, a pair of frequency extension modules (75–110 GHz), and a pair of identical standard gain horn antennas for transmission and reception. The angle between two antennas was fixed at 5°. The FAMMA device was placed on the testing platform at about 0.7 m away from each antenna.

### Application Testing Setup

For radar detection experiments, a radar system was installed in an open area with sufficient space. The effective detection range of the radar extended to 30 cm on either side and 1 m in front of the radar center. The boundaries of the effective detection zone were marked with white tapes. Afterward, a remotely controlled car model was guided to move from through the radar detection zone.

A FMCW-based radar scanner was used for radar imaging stealth testing. After the completion of system calibration, the FAMMA-based MMA device was placed on the scanning platform, and the radar transceiver sensor was software-controlled to scan and generate radar images.

### Material Properties and Condition Settings in Numerical Simulation

Finite element analysis was conducted in CST Studio Suite 2023. Unit cell boundary conditions were set in the *x* and *y* directions, while the boundary in the *z* direction was set to open. EM waves were incident from the *z* direction, and the incident port was set as Floquet port. The polarization angle was controlled by adjusting *φ*, and the incident angle was controlled by adjusting *θ* in CST.

The calculation methods for the complex dielectric constant $\varepsilon \left( \omega \right)$ and loss tangent tanδ are shown in Eqs. (1) and (2):

1 $$\begin{array}{*{20}c} {\varepsilon \left( \omega \right) = \varepsilon^{\prime } \left( \omega \right) - j\varepsilon^{\prime \prime } \left( \omega \right)} \\ \end{array}$$

2 $$\begin{array}{*{20}c} {\tan \delta = \varepsilon^{\prime \prime } \left( \omega \right)/\varepsilon^{\prime } \left( \omega \right)} \\ \end{array}$$

The refractive index $n\left( \omega \right)$ and absorption coefficient $\alpha \left( \omega \right)$ of HTL resin were derived from the transmission spectra obtained using terahertz time-domain spectroscopy (TDS) by Eqs. (3) and (4) [29–31]:

3 $$\begin{array}{*{20}c} {n\left( \omega \right) = \varphi \left( \omega \right)\frac{{c_{0} }}{d} + 1} \\ \end{array}$$

4 $$\begin{array}{*{20}c} {\alpha \left( \omega \right) = \frac{2}{d}\ln \left( {\frac{4n\left( \omega \right)}{{\rho \left( \omega \right)\left( {n\left( \omega \right) + 1} \right)^{2} }}} \right)} \\ \end{array}$$

where *φ(ω)* and *ρ(ω)* are the phase and amplitude ratios of frequency-domain signals measured in transmission mode with and without HTL resin, which is calculated by Fourier transform from the measured time-domain signal. *ω* is the angular frequency of the incident wave, *d* is the thickness of the resin, and *c*_*0*_ is the speed of light in vacuum. The real and imaginary parts of the relative complex dielectric constant of HTL resin were calculated according to Eqs. (5) and (6):

5 $$\begin{array}{*{20}c} {\varepsilon^{\prime}\left( \omega \right) = \left( {n\left( \omega \right)} \right)^{2} - \left( {\frac{{c_{0} \alpha \left( \omega \right)}}{2\omega }} \right)^{2} } \\ \end{array}$$

6 $$\begin{array}{*{20}c} {\varepsilon^{\prime\prime}\left( \omega \right) = \frac{{c_{0} n\left( \omega \right)\alpha \left( \omega \right)}}{\omega }} \\ \end{array}$$

The relative dielectric constant and loss tangent of the HTL resin were set to 2.92 and 0.02 according to measured values (Fig. S2 and Table S1), respectively. The conductivity of the aluminum foil was set to 4.561 × 10^7^ S m^−1^.

## Results and Discussion

### FAMMA Design Concept and Analysis

**Fig. 1:**
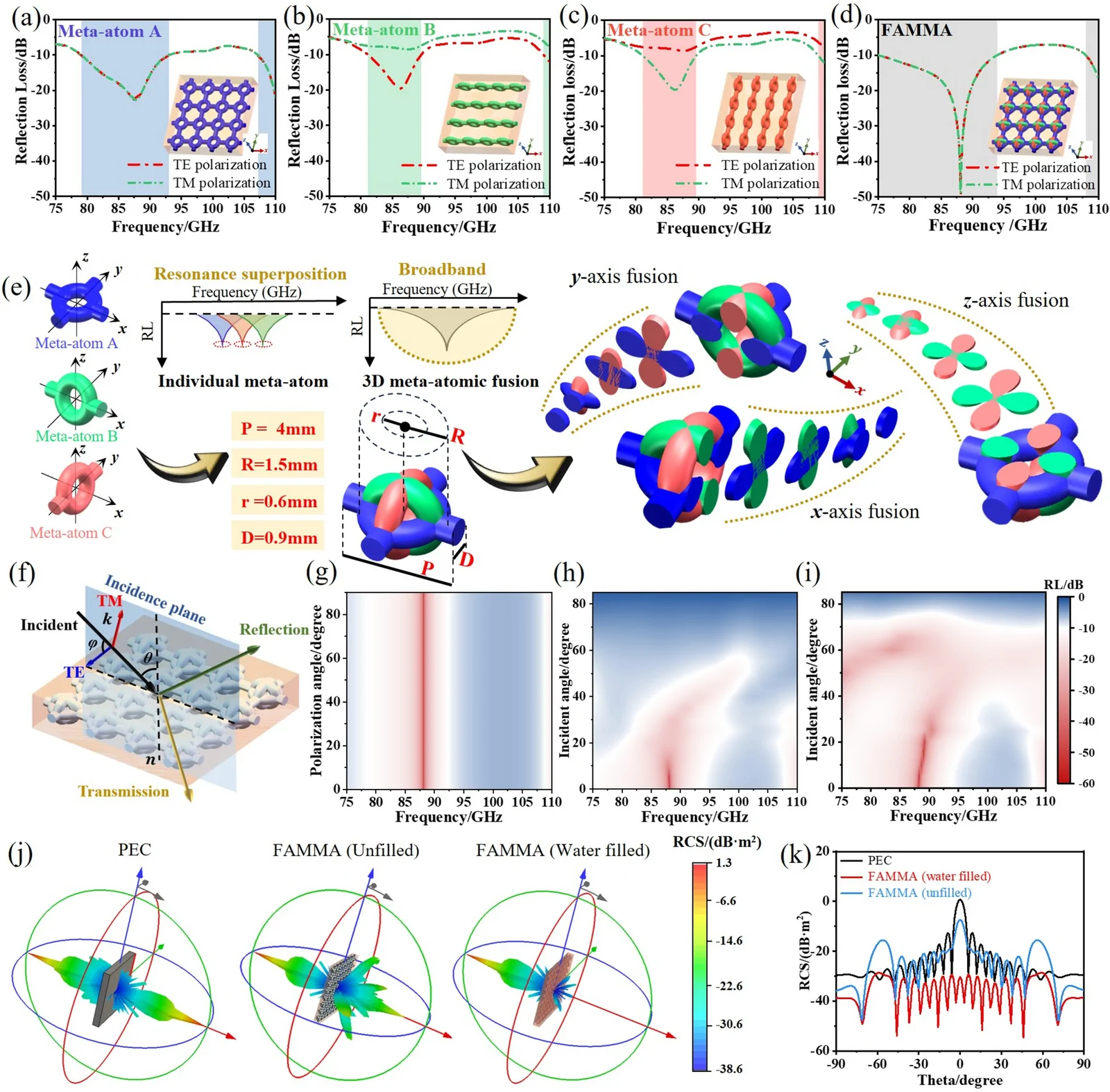
Structural schematic diagram and simulation results of: **a** Meta-atom A; **b** Meta-atom B; **c** Meta-atom C; **d** FAMMA by 3D fusion of annular microfluidic meta-atoms. **e** Schematic illustration of the concept and effect of meta-atomic fusion. **f** Schematic diagram of polarization angle and incident angle. **g** Polarization insensitivity characteristic of FAMMA. Wide-angle incidence characteristic of FAMMA under **h** TE Polarization and **i** TM Polarization. **j** Three-dimensional RCS distribution of PEC, unfilled FAMMA, and water-filled FAMMA. **k** RCS variation with incident angle

In water-based MMAs, microfluidic channels and chambers are strategically designed to confine and shape water into meta-atoms with desired EM resonance characteristics. As illustrated in Fig. 1a–c, three kinds of geometrically and material-wise identical annular water meta-atoms were designed (meta-atoms A, B, and C, respectively), resulting in distinct resonant absorption responses due to different resonant coupling behavior with incident EM waves. The dimensions of each meta-atom are set as follows: the length and width are both P = 4 mm, and the total thickness of the dielectric layer is H = 3.6 mm. The outer radius of the annular microfluidic channel is R = 1.5 mm, the inner radius is r = 0.6 mm, and the diameter of the microchannel is D = 0.9 mm. The thickness of the bottom anti-transmission aluminum film is set to 1 μm (far greater than skin depth at operating frequency). The EMW absorption capacity is usually described by RL, which can be calculated according to transport theory as Eqs. (7) and (8) [32, 33]:

7 $$\begin{array}{*{20}c} {Z_{in} = Z_{0} \sqrt {\frac{{\mu_{r} }}{{\varepsilon_{r} }}} \tanh \left( {\frac{2\pi jfd}{c}\sqrt {\mu_{r} \varepsilon_{r} } } \right)} \\ \end{array}$$

8 $$\begin{array}{*{20}c} {RL = 20\log_{10} \left| {\frac{{Z_{in} - Z_{0} }}{{Z_{in} + Z_{0} }}} \right|} \\ \end{array}$$

where *Z*_*in*_ is the input impedance of sample, *Z*_*0*_ refers to the impedance of free space, *f* is frequency, *d* is the thickness, and *c* is the velocity of light. ${\mu }_{r}$ and ${\varepsilon }_{r}$ are complex permeability and complex permittivity, respectively. An RL value less than − 10 dB means that more than 90% of the EM waves are absorbed, and the band with an RL value less than -10 dB is considered an effective absorption bandwidth. Specifically, meta-atom A achieves an RL below -10 dB across 79.0–92.9 and 107.2–110 GHz (blue shaded region), with a peak minimal reflection loss (RL_min_) of − 22.7 dB at 87.7 GHz and an EAB of 16.7 GHz (Fig. 1a). Notably, meta-atom A also exhibits strong polarization insensitivity, as evidenced by the overlapping RL curves of transverse electric (TE) and transverse magnetic (TM) polarizations. By contrast, meta-atom B exhibits a strong polarization dependence (Fig. 1b). While it achieves RL below -10 dB across 81.1–89.5 and 108.9–110 GHz for TE polarization (green shaded region), its RL for TM polarization remains weak within the target band. Conversely, meta-atom C exhibits the opposite behavior, showing strong absorption of TM polarization (red shaded region) and weak absorption of TE polarization (Fig. 1c).

Previous strategies to expand the bandwidth of water-based MMAs have typically involved doping small meta-atoms into the interstitial space of lattice between large meta-atoms [22]. While effective for planar meta-atoms of distinct sizes, this approach becomes inefficient for 3D meta-atoms of similar scale due to packing steric constraints. In contrast, we introduce a novel meta-atom design by orthogonally fusing three kinds of annular microfluidic meta-atoms, into a 3D meta-atom termed FAMMA (Fig. 1d). This meta-atomic fusion can be analogized to volumetric stacking of distinct resonant units along each axis (Fig. 1e). As a result, FAMMA significantly broadens both the width and depth of the absorption band through resonance peak superposition, achieving broadband absorption across 75–93.9 and 107.9–110 GHz (gray shaded region), increasing EAB from 16.7 to 20.9 GHz, an enhancement of 25.1%. Simultaneously, the RL_min_ improves from − 22.7 to − 49.0 dB, a remarkable enhancement of 115.2% (Fig. 1d). Furthermore, the orthogonal 3D meta-atomic fusion imparts rotational symmetry to FAMMA and effectively eliminates the polarization sensitivity issue observed in meta-atoms B and C, substantially extending the applicability of FAMMA.

FAMMA also exhibits excellent polarization insensitivity and wide incident angle adaptability. The transverse electric (TE) and transverse magnetic (TM) wave polarizations are defined relative to the plane of incidence, which is formed by the wave vector (*k*) of the incident plane wave and the surface normal vector (*n*) of the metamaterial surface (Fig. 1f). The angle between the electric field component of the incident wave and the incident plane is defined as *φ*. A wave is defined as TE-polarized when *φ* = 90°with the electric field perpendicular to the plane of incidence, and TM-polarized when *φ* = 0° with the electrical field parallel to the plane of incidence. As shown in Fig. 1g, the RL curve of FAMMA remains virtually unchanged as the polarization angle of the vertically incident EM wave varies from 0° to 90°, owing to the rotational symmetry of FAMMA. Under normal incidence, both TE and TM components of incident EM waves are parallel to the device surface. Due to the rotational symmetry of FAMMA, it responses identically to these two orthogonal eigenstates of the incident EM waves, thereby eliminating the dependence of MMAs on the polarization angle of incident EM waves. FAMMA also shows wide incident angle adaptability, as shown in Fig. 1h, i. FAMMA maintains strong absorption of TE-polarized EM waves up to an incident angle of 55°, although the EAB gradually narrows as the incident angle increases. The absorption of TM-polarized EM waves by FAMMA remains strong for incident angles up to 70°, with the EAB covering the entire W-band as incident angle increases from 40° to 70°. The superior incident angle adaptability of TM polarization is attributed to different impedance scaling rate of TE- and TM-polarized waves with incident angles. The impedance for TE polarization scales proportionally with 1/cos*θ*, where *θ* is the incident angle. In contrast, impedance for TM polarization scales proportionally with cos*θ*. Since the latter varies more gradually with increase in incident angle, impedance matching is better maintained for TM polarization, resulting in strong absorption over a wide range of incident angles [34].

In order to evaluate potential radar stealth capability of FAMMA in realistic scenario, the far-field radar cross section (RCS) distribution of reflected signals was simulated (Fig. 1j). For a comparative analysis, three structures are modeled: a perfect electric conductor, the FAMMA without water filling, and the FAMMA with water filling. All simulations are performed at 88.12 GHz, and the boundary conditions are set as open in all directions, with the EMW propagating along the negative y-axis and the electric field polarized along the z-axis. The scattering direction is defined in a spherical coordinate by the angles *θ* and *φ*. The RCS of the three samples is calculated using the following Eq. (9) [35, 36]:

9 $$\begin{array}{*{20}c} {\sigma \left( {dBm^{2} } \right) = 10\log \left( {\frac{4\pi S}{{\lambda^{2} }}\left| {\frac{{E_{s} }}{{E_{i} }}} \right|^{2} } \right)} \\ \end{array}$$

where *S* is the area of the simulated sample, *λ* is the wavelength of the incident EMW, and *E*_*s*_ and *E*_*i*_ are the electric field strengths of the scattering and incident waves, respectively. The radar reflection intensity showed significantly reduced backscattering from -38.6 to 1.35 dB m^−2^ for the water-filled FAMMA. The results indicated significantly stronger attenuation of radar echo for the water-filled FAMMA compared to other two counterparts (Fig. 1k). Under normal incident (*θ* = 0°), the vertical radar scattering dropped from 0.65 dB m^2^ (PEC) and -7.41 dB m^2^ (unfilled FAMMA) to -30.38 dB m^2^ (water-filled FAMMA), highlighting the strong EM wave attenuation of FAMMA-based MMA.

### Numerical Analysis of EM Absorption Mechanism of FAMMA

**Fig. 2:**
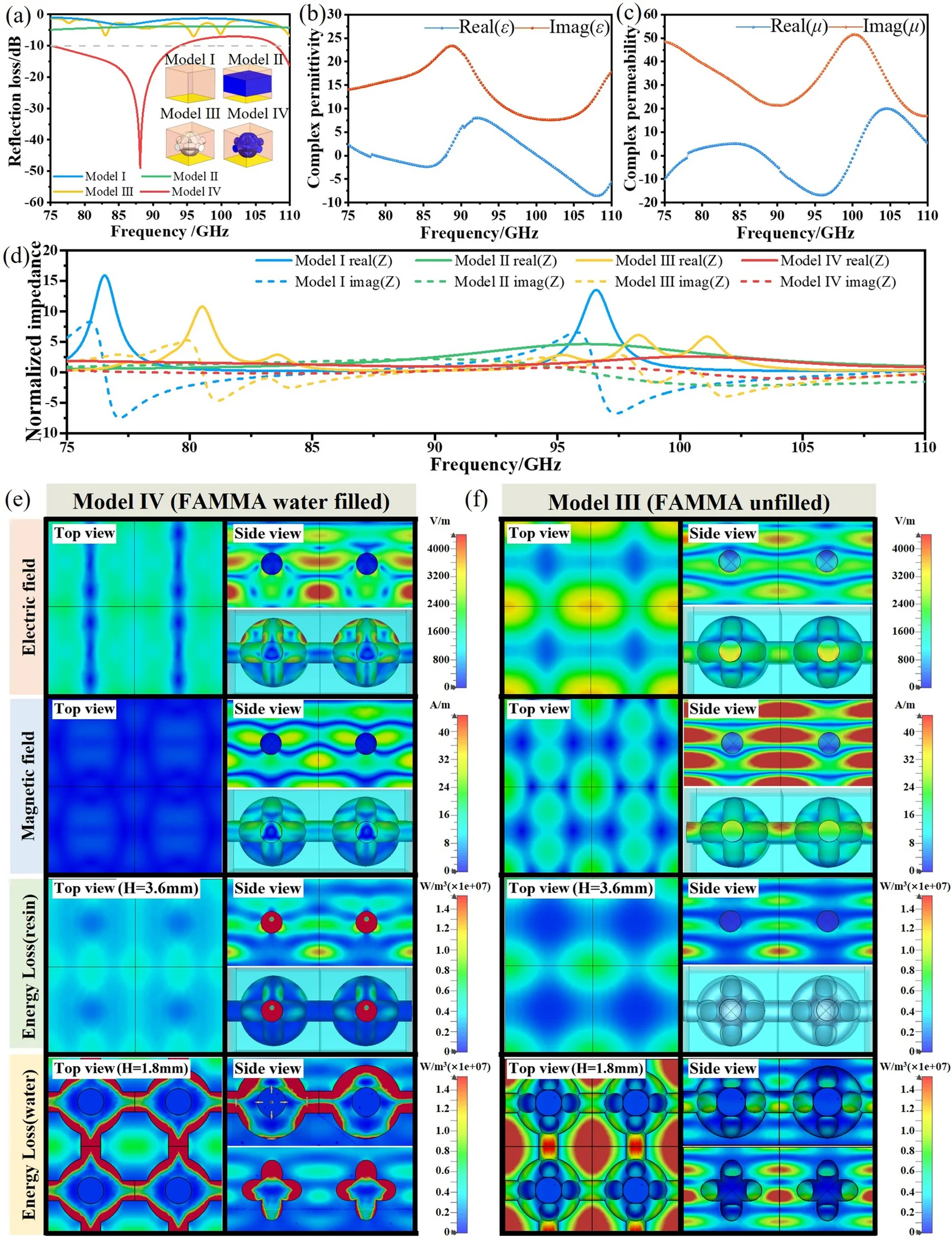
**a** Schematic diagrams of four different models used to validate the effectiveness of the synergistic solid–liquid coupling design and their RL profile. The equivalent EM parameters of FAMMA calculated through *S*-parameter inversion: **b** complex permittivity and **c** complex permeability. **d** Normalized impedance of the four models, showing both the real and imaginary components across the W-band. The electric field, magnetic field, and energy loss distribution at 88.12 GHz of **e** Model IV and **f** Model III. The top view of energy loss is at the top layer of FAMMA with H = 3.6 mm and the internal section with H = 1.8 mm, respectively. Each figure consists of an array of 2 × 2 FAMMA units, with the left subpanel showing the surface distribution (top view), and the right subpanel showing the side view and internal field distribution within the water-filled annular meta-atoms

To isolate the contribution of structure and material on the absorption performance of FAMMA, four distinct models were created for comparison (Fig. 2a)

. Model I consists of an all-dielectric 3D-printed monolithic resin block; Model II incorporates a planar water layer embedded within an identical block; Model III represents an unfilled FAMMA; and Model IV corresponds to the water-filled FAMMA. The simulated RL of Model I and Model II remains above -5 dB across the target frequency band, implying that neither the resin nor water alone (without the meta-structure) provides effective EM wave absorption. Model III, which retains the structural features of FAMMA but lacks the water, exhibits weak EM absorption with three minor resonance peaks at 83.01, 95.93, and 99.88 GHz. However, none of them falls below the -10 dB threshold typically required for efficient absorption. In stark contrast, Model IV (FAMMA) exhibits significantly enhanced broadband EM wave absorption, with an RL_min_ dipping to approximately -50 dB. This superior strong broadband absorption of FAMMA arises from coupling effect between the orthogonal water ring and the resin structure.

A classic framework employed to understand the absorption mechanism of MMA involves impedance matching analysis [37]. Perfect absorption occurs when the impedance of the MMA matches that of free space (*i.e.,* Re(Z) = 1 and Im(Z) = 0), an essential condition for perfect absorption [38]. Appropriate EM parameters can greatly improve impedance matching and further enhance EM absorption. The equivalent EM parameters of FAMMA are closely related to its relative impedance, including the real part (*ε'*) and imaginary part (*ε''*) of the complex permittivity, as well as the real part (*μ'*) and imaginary part (*μ"*) of the complex permeability [39, 40]. The relationship between them is shown in Eqs. (10) and (11) [41, 42]:

10 $$\begin{array}{*{20}c} {\varepsilon_{eff} = n/z = \varepsilon^{\prime } + j\varepsilon^{\prime \prime } } \\ \end{array}$$

11 $$\begin{array}{*{20}c} {\mu_{eff} = nz = \mu^{\prime } + j\mu^{\prime \prime } } \\ \end{array}$$

We use the *S*-parameter inversion method to calculate the equivalent EM parameters of FAMMA as a whole, including complex permittivity (*ε*_*eff*_) and complex permeability (*μ*_*eff*_). Based on the *S*-parameters (*S*_11_, *S*_21_) of FAMMA, the complex refractive index (*n*) is calculated using Eq. (12), and the normalized impedance of FAMMA is computed from the simulated *S*-parameters using Eq. (13) [43]:

12 $$\begin{array}{*{20}c} {n = \frac{1}{kd}cos^{ - 1} \left[ {\frac{1}{{2S_{11} }}\left( {1 - S_{11}^{2} + S_{21}^{2} } \right)} \right]} \\ \end{array}$$

13 $$\begin{array}{*{20}c} {Z = \sqrt {\frac{{(1 + S_{11} )^{2} - S_{21}^{2} }}{{(1 - S_{11} )^{2} - S_{21}^{2} }}} } \\ \end{array}$$

where *d* is the thickness of FAMMA, *k* is the wavenumber (*k* = *2πf*), and *c* is the speed of light. The calculated equivalent EM parameters of FAMMA through *S*-parameter inversion are shown in Fig. 2b, c. The real and imaginary components of the normalized impedance for the four models are compared in Fig. 2d. Only Model IV (FAMMA) demonstrates effective impedance matching across the entire target band, with its real component approaching 1 (red solid line) and imaginary component approaching 0 (red dash line), substantially outperforming the other three designs. This superior impedance matching [44] again highlights that the synergistic effect of FAMMA’s 3D-fused annular meta-structure and water-based lossy dielectric medium is critical for high-performance EM wave absorption.

To further elucidate the absorption mechanism of FAMMA, full-wave simulations of the EM field and energy dissipation [45] were conducted at a representative resonant frequency of 88.12 GHz. As shown in Fig. 2e, the electric field within the water-filled region is primarily localized at the water–resin interface, particularly on the outer surface of the upper half of the FAMMA. Within the solid resin, the electric field is primarily localized at the junctions between adjacent FAMMAs, especially at the bottom halves. In contrast, the magnetic field shows relatively weak coupling in both the resin and water regions. The energy dissipation profile generally mirrors the electrical field pattern with highest energy loss concentrated at the water–resin interface, suggesting that absorption is dominated by electric resonance. The localized electric field enhances polarization in the dielectrics, leading to energy dissipation through dielectric relaxation process. After filling with water, the energy loss is significantly enhanced by the coupling effect between the orthogonal water ring and the resin structure, as evidenced by the significant energy dissipation within the microfluidic network. As the electric field in water is primarily localized at the outer surface of the water-filled FAMMA meta-atom, the energy is primarily dissipated in the same region. For comparison, Fig. 2f presents the electric field and energy dissipation distributions for Model III, which shares the same microfluidic network as FAMMA but not filled with water. This configuration exhibits weaker electric field localization and drastically reduced energy dissipation in spite of enhanced magnetic coupling regions. These findings further underscore the important role of water as the lossy dielectric medium in the FAMMA system, and highlight that the structural design alone is insufficient to achieve strong broadband absorption.

### Effect of Geometric Dimensions on Broadband Absorption

**Fig. 3:**
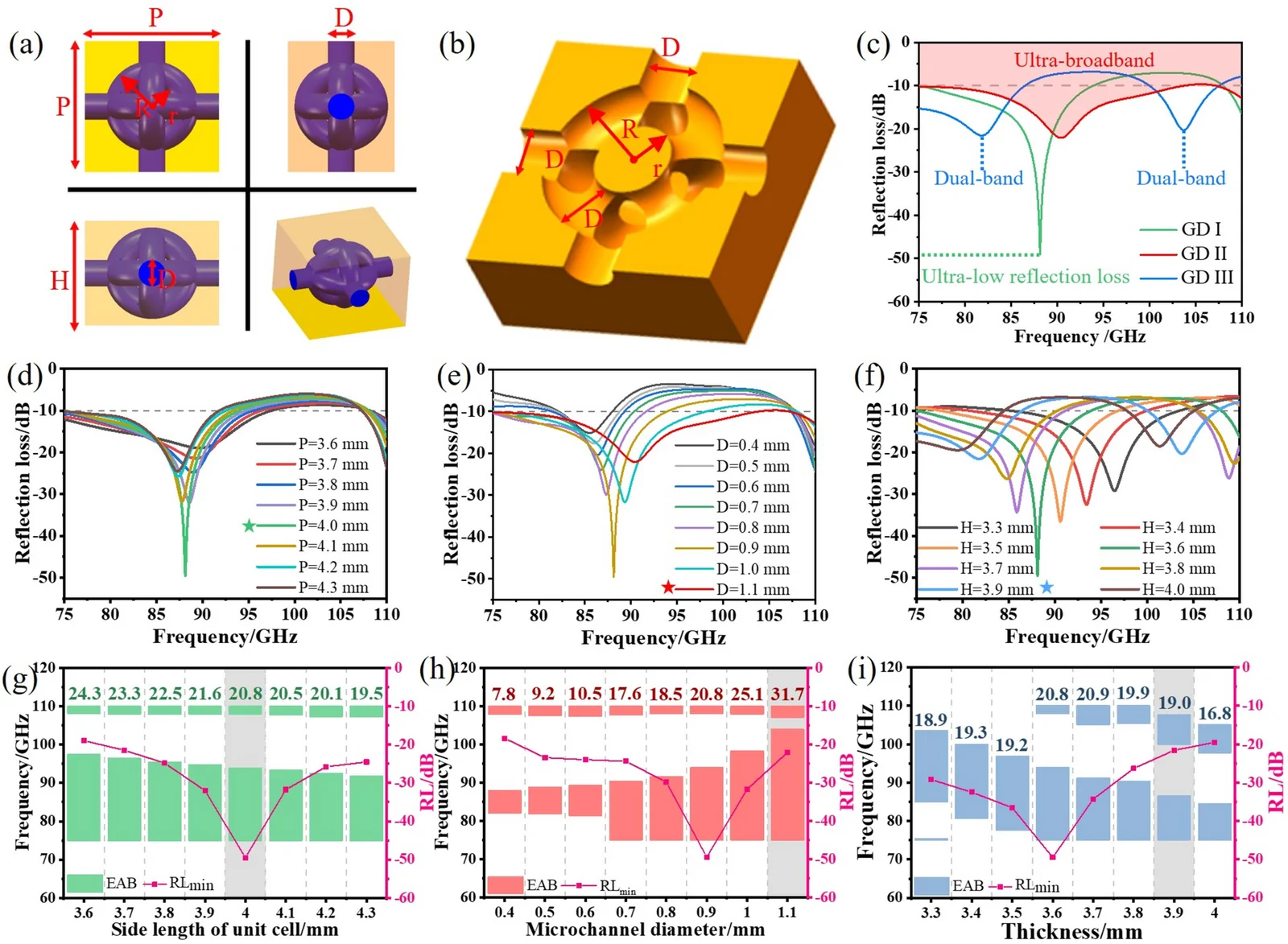
**a** Schematics of geometric dimensions and **b** cross-sectional structure of a FAMMA unit cell. **c** Representative designs GD Ⅰ, GD Ⅱ, and GD Ⅲ with their distinct RL profile. The RL profile as a function of **d** unit period *P*, **e** microchannel diameter *D*, and **f** thickness *H*. The corresponding EAB and RL_min_ are illustrated in **g**, **h**, and **i**

A complete FAMMA-based MMA is created by periodically arranging FAMMA units on top of a reflective aluminum foil to prevent EM wave transmission. The geometric dimensions (GD) of the FAMMA have a critical impact on its absorption characteristics. Key GD parameters include the overall device thickness (*H*), the FAMMA unit cell period (*P*), and the inner diameter (*r*) and outer diameter (*R*) of the annular microfluidic channel, which together define the channel width *D* = *R-r* (Fig. 3a, b). A parametric sweep is conducted on *H*, *P*, and *D* to systematically examine how these GD parameters affect the EM absorption performance of MMAs in terms of their RL, RL_min_, and EAB. As shown in Fig. 3d, the RL peak gradually shifts toward lower frequencies as *P* increases while other parameters are held constant, with RL_min_ reaching its lowest point of ~ 50 dB at *P* = 4.0 mm. However, further increase in *P* leads to weaker RL_min_ values while the peak position continues shifting toward the lower frequencies. The corresponding EAB, represented by the length of the green bar in Fig. 3g, displays a gradual decrease with increase in *P*. Similarly, the absorption characteristics varies with *D* as shown in Fig. 3e, h. As D increases, the RL peak gradually shifts toward higher frequencies, with RL_min_ reaching its strongest absorption at *D* = 0.9 mm. Notably, the EAB also increases with increase in *D*, reaching 31.78 GHz at *D* = 1.1 mm and covering the entire W-band. The device thickness *H* also influences the absorption characteristics of FAMMA as shown in Fig. 3f, i. The RL peak gradually shifts toward lower frequencies with increase in *H*, with RL_min_ reaching its strongest absorption at *H* = 3.6 mm. As *H* further increases beyond this point, the EAB band gradually bifurcates into two distinct bands of comparable bandwidth while maintaining a similar level of total EAB.

Based on the analysis above, three representative GD designs with unique absorption characteristics were selected for further evaluation (Fig. 3c). The FAMMA-based MMA device with geometric dimension Ⅰ (GD Ⅰ, *P* = 4.0 mm, *H* = 3.6 mm, *D* = 0.9 mm, *r* = 0.6 mm, *R* = 1.5 mm) achieves ultra-strong absorption with an ultra-low RL_min_ of approximately -50 dB and a broad EAB of 20.8 GHz; the device with GD Ⅱ (*P* = 4.0 mm, *H* = 3.6 mm, *D* = 1.1 mm, *r* = 0.6 mm, *R* = 1.7 mm) exhibits relatively weak absorption with RL_min_ of approximately -20 dB but an ultra-broadband EAB covering almost the entire W-band; the device with GD Ⅲ (*P* = 4.0 mm, *H* = 3.9 mm, *D* = 1.1 mm, *r* = 0.6 mm, *R* = 1.5 mm) exhibits a dual-band absorption profile, with strong absorption in both the high-frequency region (100.02–107.62 GHz) and low-frequency region (75–86.48 GHz).

### Experimental Evaluation of FAMMA Performance

**Fig. 4:**
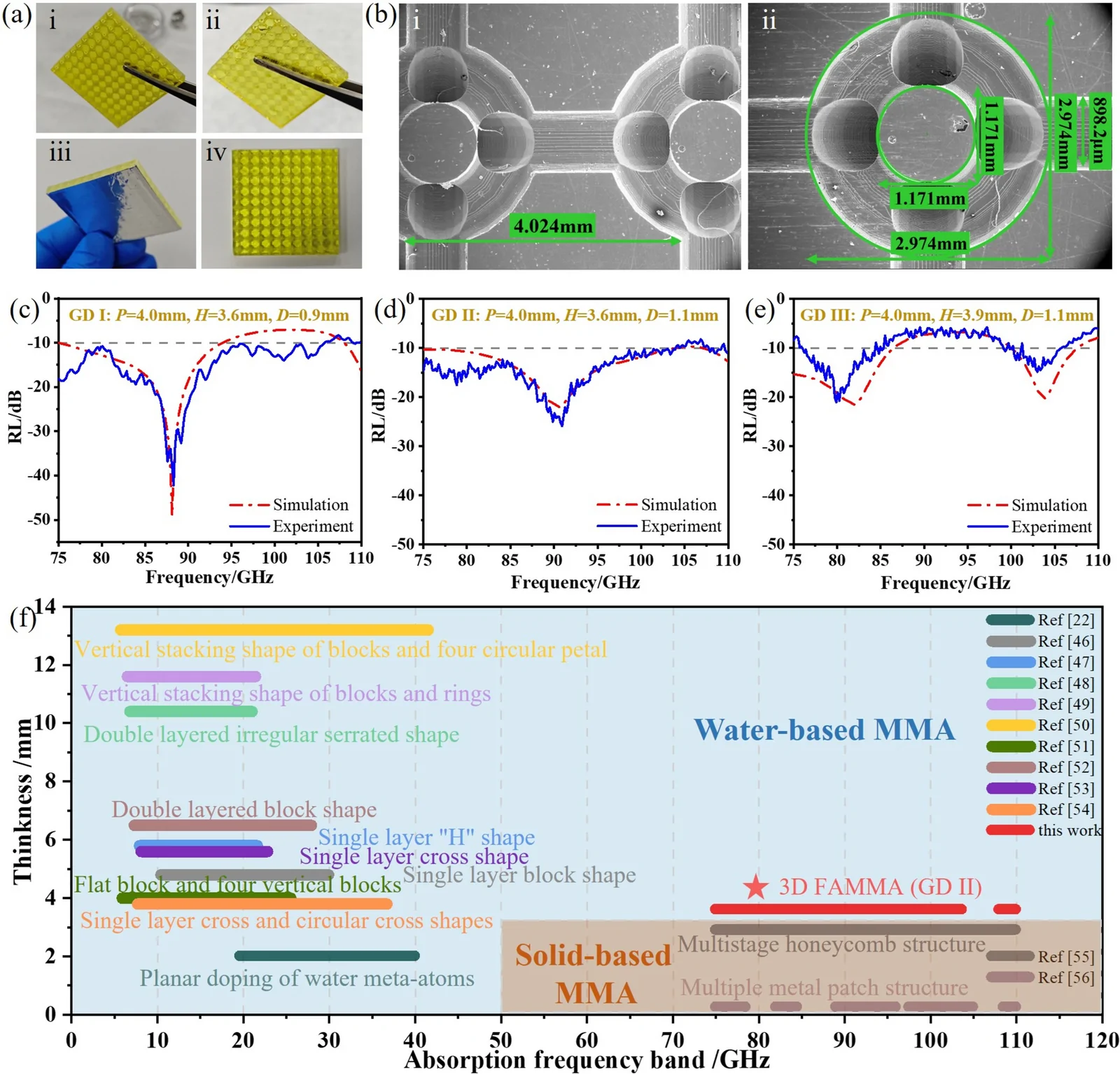
**a** Pictures of FAMMA. (i) 3D-printed FAMMA as is, (ii) water-filled FAMMA, (iii) attachment of aluminum foil as reflective layer at the bottom, (iv) FAMMA with inlets sealed. **b** Characterization of FAMMA structure by SEM: **i** adjacent FAMMA unit cells, (ii) sectional view on a horizontal plane. Comparison between simulated and measured RF profiles for **c** GD I, **d** GD II, and **e** GD III. **f** Benchmark comparison of this work (red star) against previously reported water-based MMAs (blue shadow) and solid-based MMA in W-band (orange shadow), in terms of structure, thickness, and EAB

The FAMMA-based MMA was fabricated by using projection micro stereolithography (PμSL), a high-resolution form of digital light processing (DLP) 3D printing technology. Following 3D printing, the device was filled with water as the lossy dielectric medium through vacuum-assisted infusion. The device has an overall dimension of 4.0 mm × 4.0 mm × 3.6 mm (L × W × H) and comprises a 10 × 10 array of total one hundred FAMMA units (Figs. 4a and S3). SEM images of the cross section reveal the internal structure of FAMMA, confirming the successful fabrication of hollow, 3D orthogonally fused annular microchannels. The high-precision PμSL 3D printing accurately reproduces the geometric designs, with dimensional error generally < 1%, except for the inner diameter *r* of the annulus, which exhibits a slightly higher error of 2.4% (Figs. 4b and S4). This level of accuracy satisfies the requirement for EM wave absorption testing in the W-band.

The RL of the FAMMA-based MMA was measured using the free-space method in a microwave anechoic chamber, with the testing setup shown in Fig. S5. The comparisons of measured and simulated RL profiles for GD I–III are presented in Fig. 4c–e. In all cases, the measured RL profiles show a strong agreement with simulated results, validating the broadband absorption capability of FAMMA. In GD I, the measured RL_min_ reaches -42.1 dB at 88.3 GHz, realizing strong broadband absorption as designed (Fig. 4c). The measured EAB of GD II reaches 31.3 GHz, almost covering the entire W-band, and the RL profile closely matches the simulation results (Fig. 4d). GD III displays dual-band absorption with slight blue shift in the measured RL profile compared to the simulated results. The EAB located at both low-frequency (76.0–85.3 GHz) and high-frequency regions (99.1–105.6 GHz) appears slightly narrower than simulated (Fig. 4e). These deviations in resonance frequency and EAB are likely due to discrepancies between actual and ideal material properties used in the simulation, minor manufacturing inaccuracy, and inherent measurement errors of the free-space method.

Additionally, Models I–III were also experimentally evaluated, with their respective RL profile presented in Fig. S6. Consistent with the simulations, none of Model I, Model II, and Model III demonstrates effective EM wave absorption in the W-band. The RL profile of Model I and Model II remains weaker than -10 dB across the target band and appears relatively flat without any evident resonance peaks, indicating negligible EM wave absorption in the absence of meta-structures. The simulated RL profile of Model III displays three resonant peaks at 82.17, 96.52, and 106.32 GHz, respectively, closely matching the simulated locations except for the third peak, which shows a red shift of approximately 6 GHz. While the measured absorption at these resonant peaks slightly exceeds the -10 dB threshold, it is insufficient to be qualified as effective broadband absorption, further underscoring the critical role of combined meta-structure and water’s lossy dielectric properties in enabling strong and broadband absorption in FAMMA.

To explore the tunability of FAMMA-based MMA, the annular microfluidic channels were filled with alternative loss medium such as GaInSn liquid metal and MXene, and their absorption performance in the W-band was experimentally tested (Fig. S7). The liquid metal-filled FAMMA exhibits multi-band narrowband absorption, with RL peaks of − 32.5, − 29.7, − 21.8, and − 26.0 dB at 83.7, 89.1, 95.8, and 103.5 GHz, respectively. In comparison, The MXene-filled FAMMA exhibits dual-band broadband absorption in the frequency ranges of 75.4–85.0 and 95.6–110.0 GHz. Notably, unlike water-filled FAMMA, the RL at high-frequency resonance shows a stronger absorption (23.8 dB @ 102.9 GHz) compared to the lower-frequency absorption (− 17.3 dB @ 79.6 GHz).

To benchmark the performance of FAMMA, we compared its key performance metrics, including the overall thickness and EAB, with previously reported water-based MMAs [22, 26, 46–55] (Fig. 4f). Among them, FAMMA exhibits the second EAB while maintaining a compact thickness of merely 3.6 mm. In contrast, most prior water-based MMAs operate primarily below 40 GHz and often require significantly thicker structures to achieve comparable absorption performance. In experiments, the RL of FAMMA reaches below − 40 dB, closely approaching the simulated value of − 50 dB, confirming its strong absorption in the target band. This exceptional performance is attributed to the unique meta-atomic fusion strategy and high-precision micro 3D printing-enabled fabrication of intricate 3D microfluidic architectures. Together, FAMMA promises outstanding potential for radar stealth and other EM wave absorption applications.

Regarding thermal stability, we have simulated the EMW absorption performance of water-based FAMMA at different temperatures using the Debye model to describe the dielectric properties and loss characteristics of water in CST by Eq. (14):

14 $$\begin{array}{*{20}c} {\varepsilon \left( {\omega ,T} \right) = \varepsilon_{\infty } \left( T \right) + \frac{{\varepsilon_{s} \left( T \right) - \varepsilon_{\infty } \left( T \right)}}{1 - i\omega \tau \left( T \right)}} \\ \end{array}$$

where ***ε***_***s***_ is the static dielectric constant, ***ε***_***∞***_ is the optical dielectric constant, *τ* is the rotational relaxation time, and *T* is the temperature of water. The temperature-dependent parameters are defined via Eqs. (15–17):

15 $$\begin{array}{*{20}c} {\varepsilon_{s} \left( T \right) = a_{1} - b_{1} T + c_{1} T^{2} - d_{1} T^{3} } \\ \end{array}$$

16 $$\begin{array}{*{20}c} {\varepsilon_{\infty } \left( T \right) = \varepsilon_{s} \left( T \right) - a_{2} e^{{ - b_{2} T}} } \\ \end{array}$$

17 $$\begin{array}{*{20}c} {\tau \left( T \right) = c_{2} e^{{\frac{{T_{1} }}{{T + T_{0} }}}} } \\ \end{array}$$

where $$a_{1 }$$ = 87.9, $$b_{1}$$ = 0.404 K^−1^, $$c_{1}$$ = 9.59 × 10^–4^ K^−2^, $$d_{1}$$ = 1.33 × 10^–6^ K^−3^,

$$a_{2}$$ = 80.7, $$b_{2}$$ = 4.42 × 10^–3^ K^−1^, $$c_{2}$$ = 1.37 × 10^–13^ s, $$T_{0}$$ = 133 °C, $$T_{1}$$ = 651 °C.

The results show the changes of complex permittivity and complex permeability of water with temperature. The absorption properties of water-based FAMMA at different temperatures can be obtained by importing the results into CST for simulation.

Figure S8a, b shows the variation of the dielectric constant of water at different temperatures. This model accurately captures the decrease in real part and imaginary parts of water’s dielectric constant with increase in frequency. By importing the temperature-dependent dielectric constants into the simulation, the RL at different temperatures is obtained. As shown in Fig. S8c, FAMMA is largely temperature-insensitive. Although a slight change in RL is observed, the effective absorption bandwidth (with RL < 10 dB) remains almost unchanged across various temperatures.

### Applications of FAMMA to Radar Stealth

**Fig. 5:**
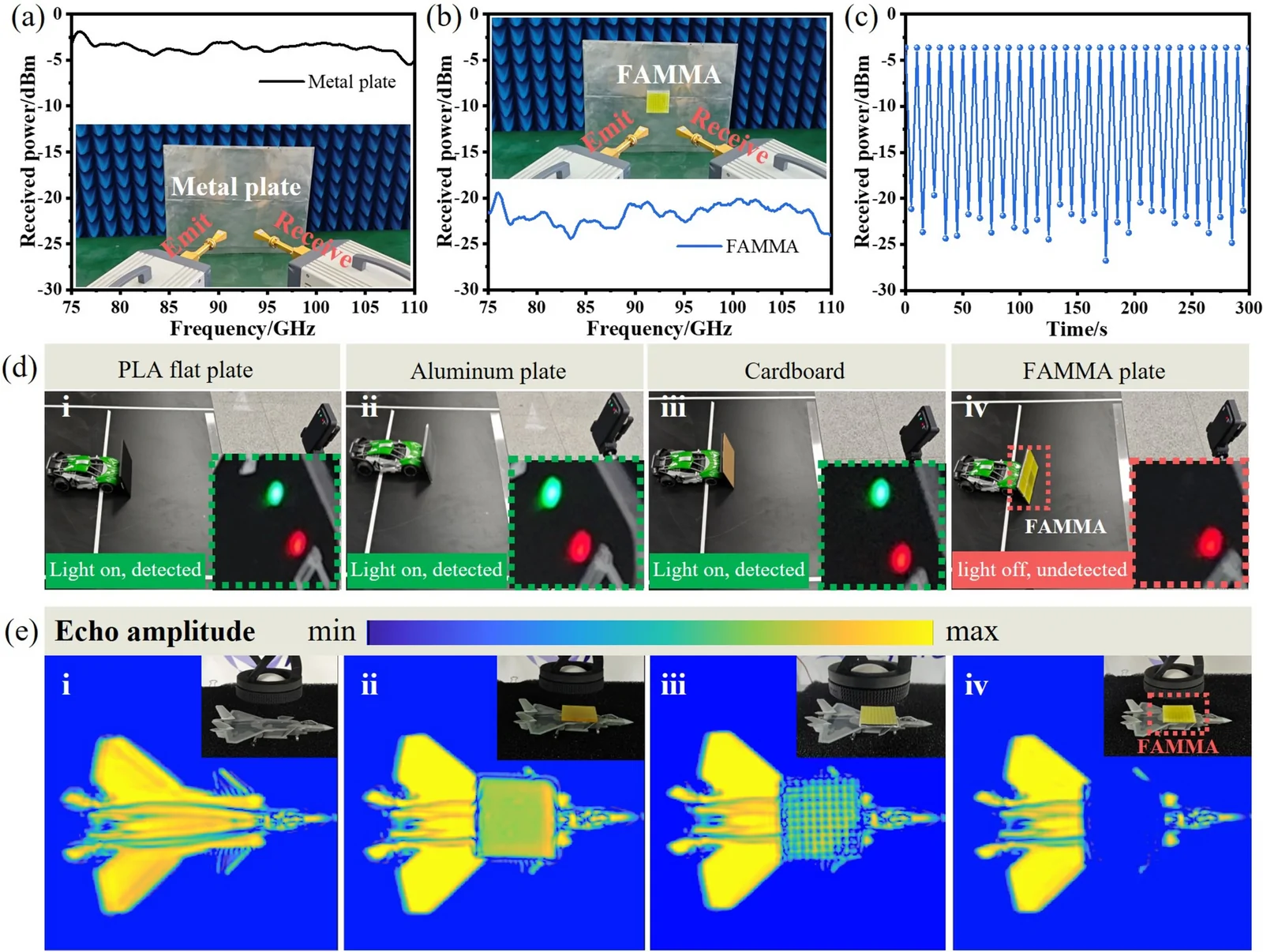
Demonstration of FAMMA’s radar stealth and echo power suppression capabilities. **a** Testing and results of reflected echo power from metal plates. **b** Significant reduction in echo power due to strong absorption by FAMMA compared to a metal plate. **c** Comparison of echo power with and without FAMMA-based MMA. **d** Application in radar stealth. (i–iii) Model car shielded with a PLA plate, aluminum foil, and cardboard results in radar detection. (iv) Model car shielded by FAMMA is not detected by the radar. **e** Radar imaging stealth demonstration. (i–iii) unshielded metal fighter jet clearly visible in radar image; (ii) fighter jet shielded with a solid resin plate remains detectable; (iii) shielding with unfilled FAMMA exhibits partial stealth; (iv) shielding with water-filled FAMMA results in complete stealth

The practical demonstrations in Fig. 5 are based on direct experimental measurements. To evaluate the potential of FAMMA in radar stealth applications, we first assessed its capability in absorbing EM energy by measuring echo power in the W-band using a vector analyzer (Fig. 5a, b). The mean reflected signal from a metal plate is -3.6 dBm, while with FAMMA, the mean reflected signal at the receiving end drops to − 21.7 dBm, corresponding to an 18.1 dBm attenuation due to EM absorption by FAMMA. In addition, repeated insertion and removal of the device further confirms effective EM wave absorption by FAMMA (Fig. 5c), showing a cyclic echo power variation between -3.6 dBm (FAMMA removed) and − 26.7 dBm (FAMMA inserted). These results validate FAMMA’s ability to effectively suppress radar reflection.

To further validate the radar stealth performance, FAMMA-based MMAs were mounted to a model car and a model fighter jet in a mock stealth scenario. The experimental setups are depicted in Fig. S9. In this setup, a radar detector is used to identify approaching objects, with a red indicator light signaling active radar operation and a green light indicating successful detection. When the model car is shielded with a polylactic acid (PLA) plate (Fig. 5di), an aluminum foil-covered plate (Fig. 5dii), or a cardboard (Fig. 5diii), the green indicator light is consistently activated, suggesting that these materials are unable to shield the object from radar detection. In contrast, when shielded with FAMMA-based MMAs, the model car moves through the radar zone undetected, effectively achieving radar invisibility (Fig. 5div and Video S1).

To provide an intuitive visualization of FAMMA’s radar stealth capability, a frequency-modulated continuous-wave (FMCW) radar imaging system (Fig. S10) was utilized to systematically compare the stealth performance of four shielding conditions: (1) unshielded metal fighter jet, (2) shielded with an all-resin solid flat plate, (3) shielded with unfilled FAMMA, and (4) shielded with water-filled FAMMA plate. As shown in Fig. 5ei, the unshielded fighter jet model is clearly visible in the radar image. When shielded with a 3D-printed solid resin plate, the plate itself becomes visible in the radar image, offering no stealth effect (Fig. 5eii). With unfilled FAMMA, partial absorption is observed, and the internal microfluidic structures of FAMMA become visible in the radar image, suggesting inadequate radar performance (Fig. 5eiii). Remarkably, once fully filled with water, FAMMA demonstrates exceptional radar stealth capability, with the shielded portion of the model fighter jet completely disappeared from the radar images, while the unshielded tail portion remains clearly visible (Fig. 5eiv). These experimental validations collectively demonstrate the superior radar stealth capability of the FAMMA-based MMA and verify its practical utility.

## Conclusions

We have developed a novel metamaterial known as FAMMA by strategically fusing 3D microfluidic annular meta-atoms. FAMMA demonstrates high-efficiency broadband absorption in the W-band. High-precision micro 3D printing technology enables accurate fabrication of FAMMA microfluidic network, which, once filled with water, achieve effective impedance matching. SEM confirms high structural fidelity and low fabrication error across critical geometric dimensions. In terms of device packaging, the tape-encapsulation method employed for FAMMA is a widely adopted and effective method for water-based MMA currently. It can effectively prevent liquid leakage, and can be well stored and used in laboratory settings. We acknowledge that developing packaging with enhanced stability for multi-scenario applications is an important objective for future research. The EM absorption and energy dissipation mechanism of FAMMA are elucidated by full-wave simulations, and the geometric design of FAMMA is optimized through parametric sweeping. The performance of FAMMA-based MMA is validated both by simulation and experimental measurements, GD I, II, and III can achieve ultra-low RL (− 42.1 dB), ultra-broadband absorption (EAB reach 31.3 GHz), and dual-band absorption (in 76.0–85.3 and 99.1–105.6 GHz), respectively. In addition, we also studied the absorption performance of FAMMA at different temperatures and incident angles through simulation, and the results showed that this design can exhibit excellent temperature insensitivity and wide incident angle characteristics. FAMMA-based MMA demonstrates excellent functionality in radar stealth applications. It significantly suppresses radar echo and prevents being captured by radar detector and FMCW radar imaging systems. By replacing water with liquid metal and MXene suspension, FAMMA-based MMA shows tunable absorption depending on the dielectric properties of the filler.

The 3D fusion FAMMA is distinguished from earlier works in two key aspects. First, unlike traditional MMA design methods with vertical stacking or horizontal planar array, our strategy leverages high-precision 3D printing to fabricate a complex three-dimensional annular microchannel structure. This intricate geometry, which was previously unattainable, enables unique solid–liquid coupling in the FAMMA. Furthermore, instead of targeting traditional microwave frequency band (0–40 GHz), offering significant potential for future applications, FAMMA is engineered for broadband absorption in the millimeter wave range (W-band).

This study introduces a unique meta-atomic fusion strategy for MMA and employs high-precision micro 3D printing technology to fabricate FAMMA with complex internal structures. The results open new avenue for realizing innovative metamaterial structures in future high-speed communication, automobile driver-assistance systems, through-wall sensing, and drone detection**.**

## Supplementary Information

Below is the link to the electronic supplementary material.

Supplementary file1 (DOCX 9471 KB)

Supplementary file2 (MP4 5098 KB)
