# Supplementary material for: Annular Microfluidic Meta-Atom Fusion-Enabled Broadband Metamaterial Absorber
Source: Nanomicro Lett. 2026 Jan 5;18:169. doi: 10.1007/s40820-025-02018-2 (PMC12765792; doi:10.1007/s40820-025-02018-2)
Supplement: Supplementary file 1 — Supplementary file1 (DOCX 9471 KB) [file 40820_2025_2018_MOESM1_ESM.docx]

Supporting Information for

**Annular Microfluidic Meta-Atom Fusion-Enabled Broadband Metamaterial Absorber**

Jinpeng Peng^1†^, Yi Zhang^1,2†*^, Zihao Chen^3^, Qiye Wen^3^, Shaomeng Wang^3^, Yaoyao Li^1^, Aiwu Zhou^1^, Zhengnan Sun^1^, Xiaohui Mu^1^, Xiaosheng Zhang^1*^

^1^ School of Integrated Circuit Science and Engineering, University of Electronic Science and Technology of China, Chengdu 611731, P. R. China

^2^ Tianfu Jiangxi Laboratory, Chengdu 641419, P. R. China

^3^ School of Electronic Science and Engineering, University of Electronic Science and Technology of China, Chengdu 611731, P. R. China

^†^Jinpeng Peng and Yi Zhang contributed equally to this work.

^*^Corresponding authors. E-mail: [zhangxs@uestc.edu.cn](mailto:zhangxs@uestc.edu.cn) (Xiaosheng Zhang); [yi_zhang@uestc.edu.cn](mailto:yi_zhang@uestc.edu.cn) (Yi Zhang)

**Supplementary Tables and Figures**

**Table S1** Dielectric constant of photocurable resins in reported water-based MMAs

| Resin type | dielectric constant | loss tangent | Design frequency band | Verified through experiment |
| --- | --- | --- | --- | --- |
| Photosensitive resin [S1] | 2.9-j0.058 | 0.02 | 15-100 GHz | No |
| Photosensitive resin [S2] | 3.4-j0.2 | 0.058 | 4-28 GHz | No |
| Photosensitive resin [S3] | 3.5-j0.35 | 0.1 | 5-30 GHz | No |
| HTL resin from BMF [S4] | 2.85-j0.22 | 0.078 | 1 THz | Yes |
| HTL resin from BMF [S5] | 2.8-j0.224 | 0.08 | 1 THz | Yes |
| BIO resin from BMF [S6] | 2.667-j0.122 | 0.046 | 1 THz | Yes |


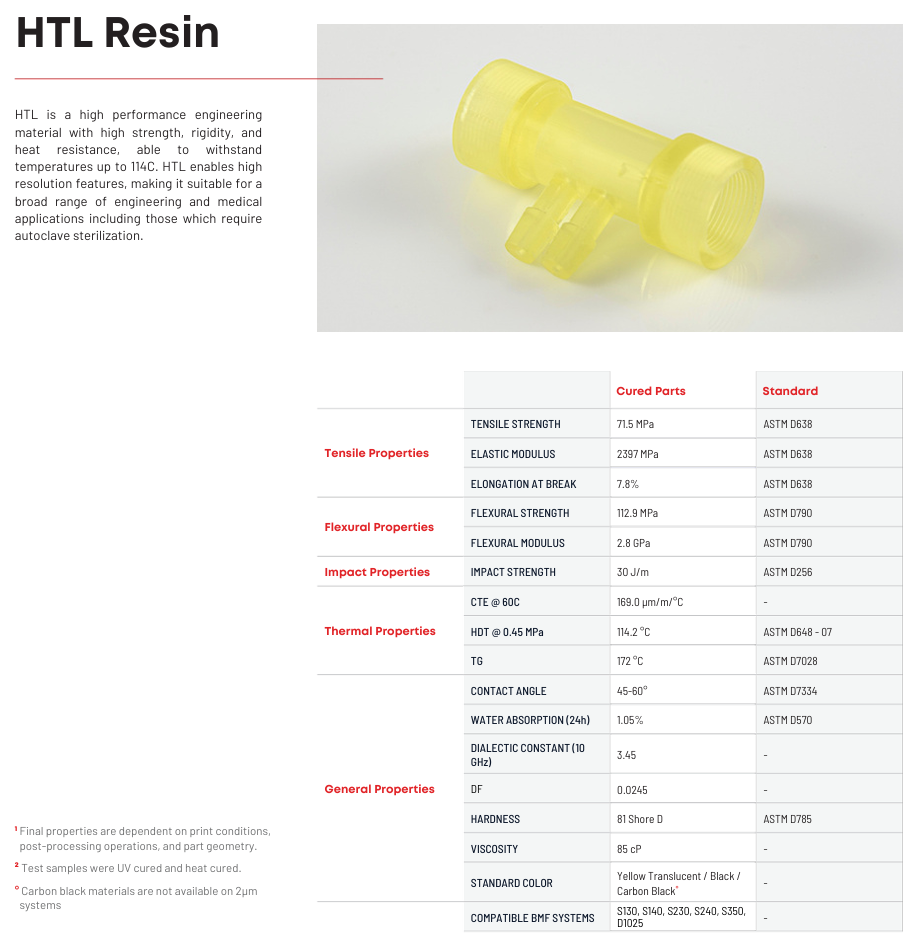


**Fig. S1** Mechanical performance parameters of HTL resin

The FAMMA structures in this work are fabricated with a PμSL 3D printer (MicroArch S240, Boston Microfabrication BMF) using their standard HTL resin. This commercial photosensitive resin has a well-characterized tensile strength of 72 MPa, an elongation at break of 8%, an elastic modulus of 2.5 GPa, a bending strength of 113 MPa, a bending modulus of 2.8 GPa, and a Shore hardness of 81D. More can be accessed through the following links: [Materials - Boston Micro Fabrication - 3D Printing Resins : BMF Boston Micro Fabrication](https://bmf3d.com/micro-3d-printing-materials/).


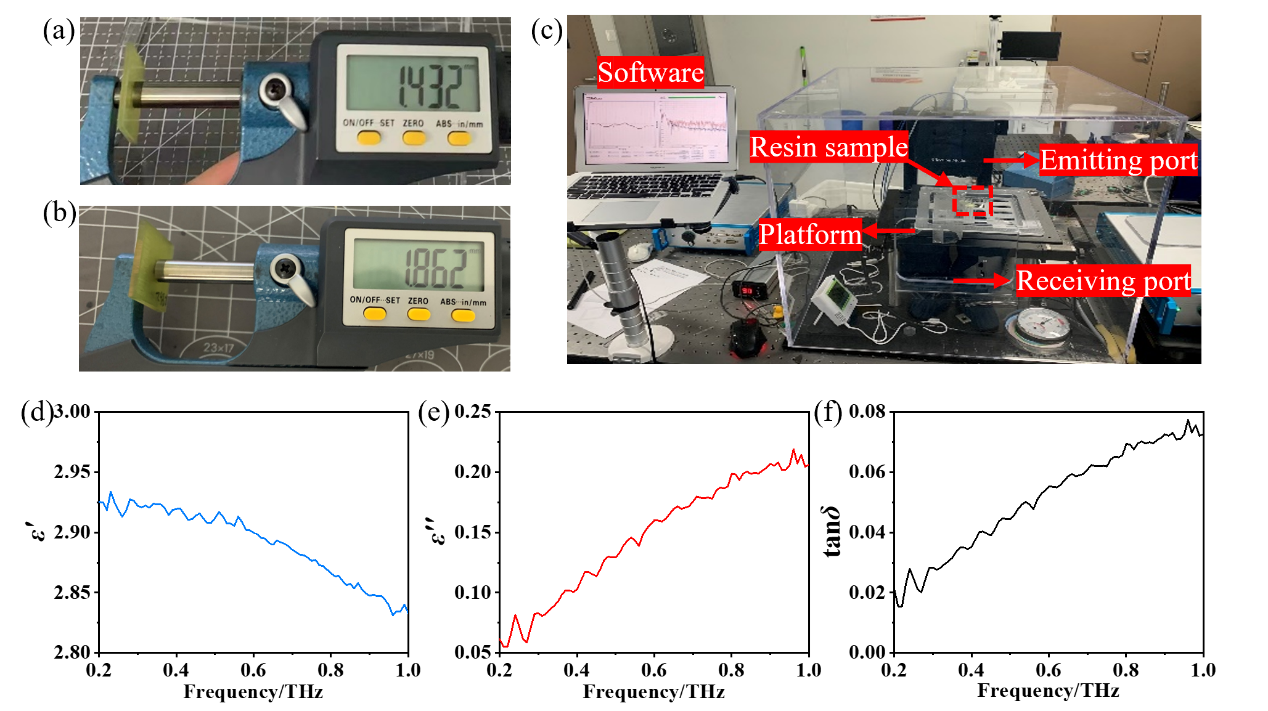


**Fig. S2** Measurement of electromagnetic parameters of HTL resin. (**a-b**) 3D-printed specimens of different thicknesses; (**c**) Transmission test results using terahertz time-domain spectroscopy, showing the: (**d**) average real part of the dielectric constant; (**e**) average imaginary part of the dielectric constant; (**f**) loss tangent

The dielectric constant of the material is a key parameter for EM simulation in CST software. However, values reported in the literatures—typically measured around 1 THz—is unsuitable for simulation in W band due to the large frequency gap. Therefore, in this study, Terahertz Time Domain Spectroscopy (TDS) was employed to obtain the dielectric constants of HTL resin the W band by measuring the complex permittivity of two 3D-printed HTL blocks with different thicknesses.

The two HTL blocks were designed with thicknesses of 1.4 mm and 1.8 mm, and the actual measured thicknesses were1.432 mm and 1.862 mm. The two specimens were subjected to TDS transmission tests. The collected time-domain signals were Fourier transformed to calculate the real and imaginary dielectric constant and loss tangent of HTL resin. According to the measured values, the dielectric constant of the resin used was set to 2.92, the loss tangent was set to 0.02, and the frequency point was set to 200 GHz in CST simulation. Although the measurement was conducted at approximately 200 GHz, CST automatically extrapolates the dielectric constants according to the target frequency bands during simulation.


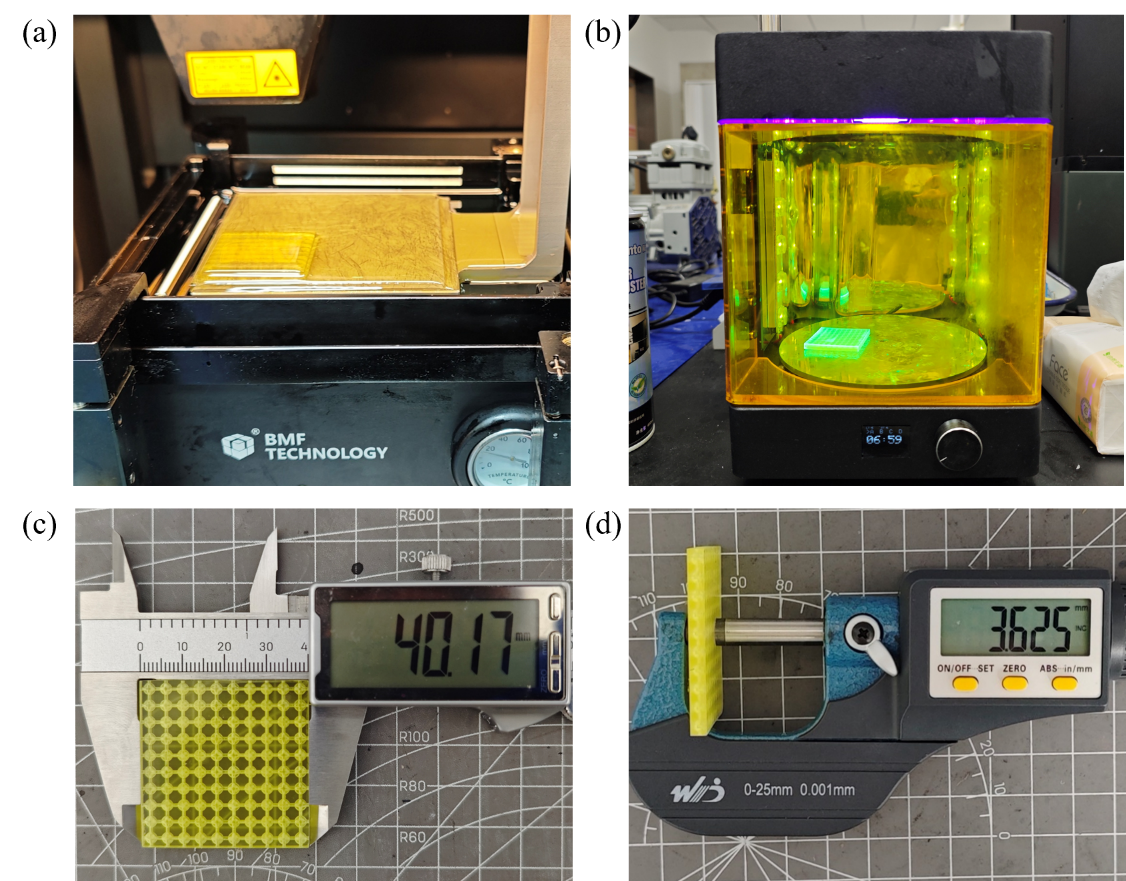


**Fig. S3** 3D printing of FAMMA. (**a**) Fabrication of FAMMA-based MMA using PμSL, a high-precision 3D printing technology. (**b**) Post-printing treatment via UV irradiation for additional curing. Measurements of overall dimensions of the FAMMA-based MMA with a 10×10 array: (**c**) Side length. (**d**) Thickness


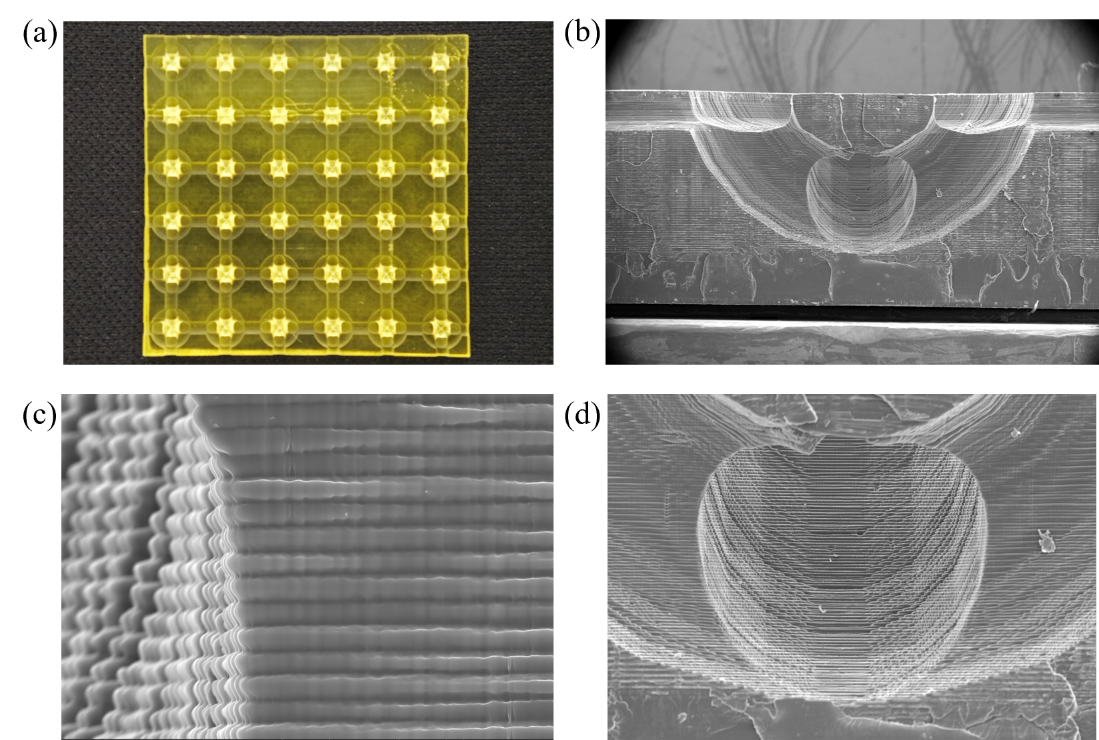


**Fig. S4** SEM characterization of FAMMA. (**a**) Cross-sectional image of the midplane of the device. (**b**) Vertical section diagram of the device. (**c**) Illustration of the layer-by-layer solidification by DLP. (**d**) SEM image of internal structure of microchannels

The printing quality and three-dimensional accuracy of microchannels were characterized by using SEM. From the results of **Figs. S3** and **S4**, the overall side length of the device is 40.17 mm vs a design value of 40 mm, with an error of 0.42%. The measured thickness H of the device is 3.62 mm vs a design size of 3.6 mm with an error of 0.69%. The measured diameter D of the microchannel is 898.2 μm vs a design value of 900 μm with an error of 0.2%. The measured side length P of the unit structure is 4.02 mm vs a designed value of 4 mm with an error percentage of 0.6%. The measured inner diameter of the annulus is 1.17 mm vs a design value of 1.2 mm with an error of 2.41%; The measured outer diameter of the annulus was 2.97 mm vs a design value of 3 mm with an error of 0.86%. All deviations fall within a narrow range, confirming the accuracy of the design.


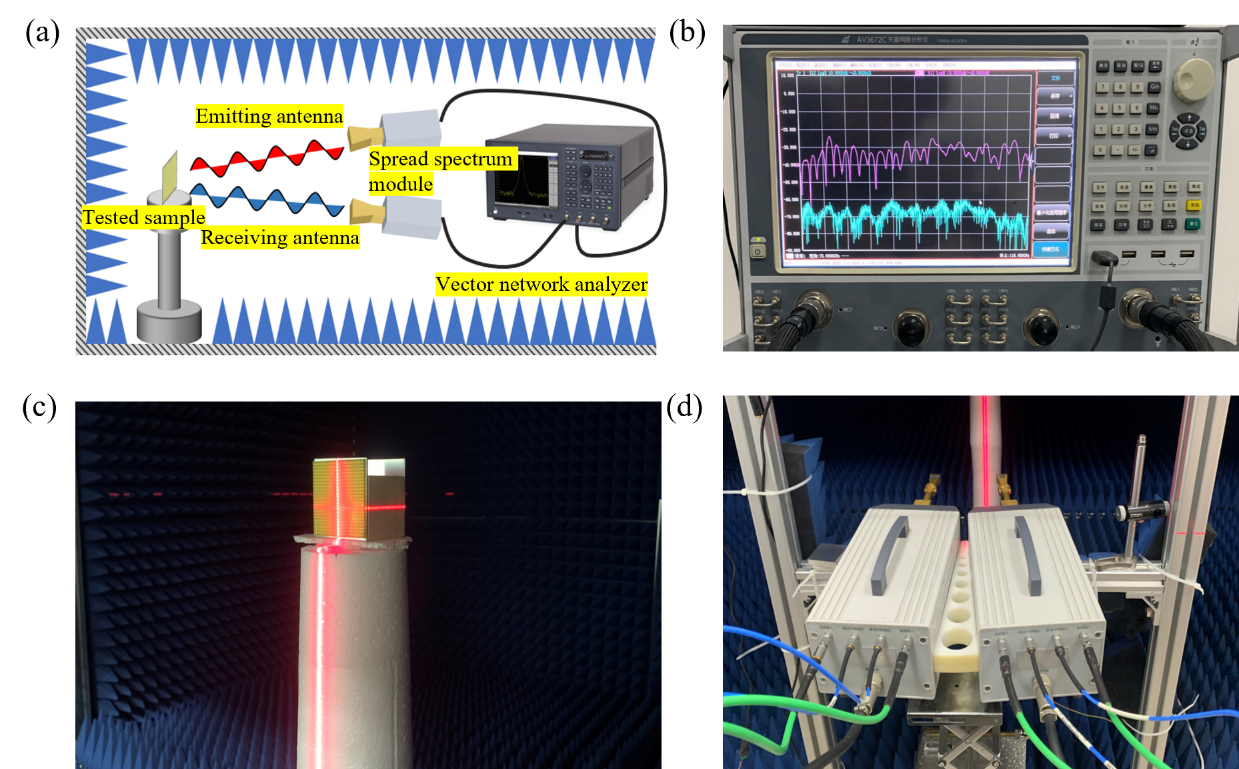


**Fig. S5** Free space method for reflection loss (RL) testing. (**a**) Schematic illustration of experiment setup. (**b**) Vector network analyzer used for signal analysis. (**c**) Placement of FAMMA-based MMA inside a microwave anechoic chamber. (**d**) Frequency extension module and horn antenna for transmitting and receiving EM wave


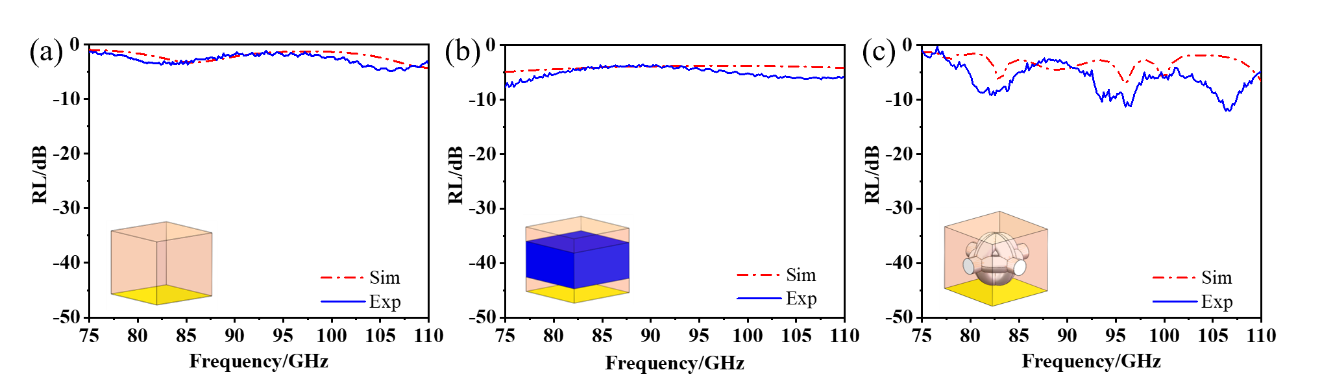


**Fig. S6** Electromagnetic absorption performance of Model I-III. (**a**) Comparison of simulated and measured RL of Model I. (**b**) Comparison of simulated and measured RL of Model II. (**c**) Comparison of simulated and measured RL of Model III

**
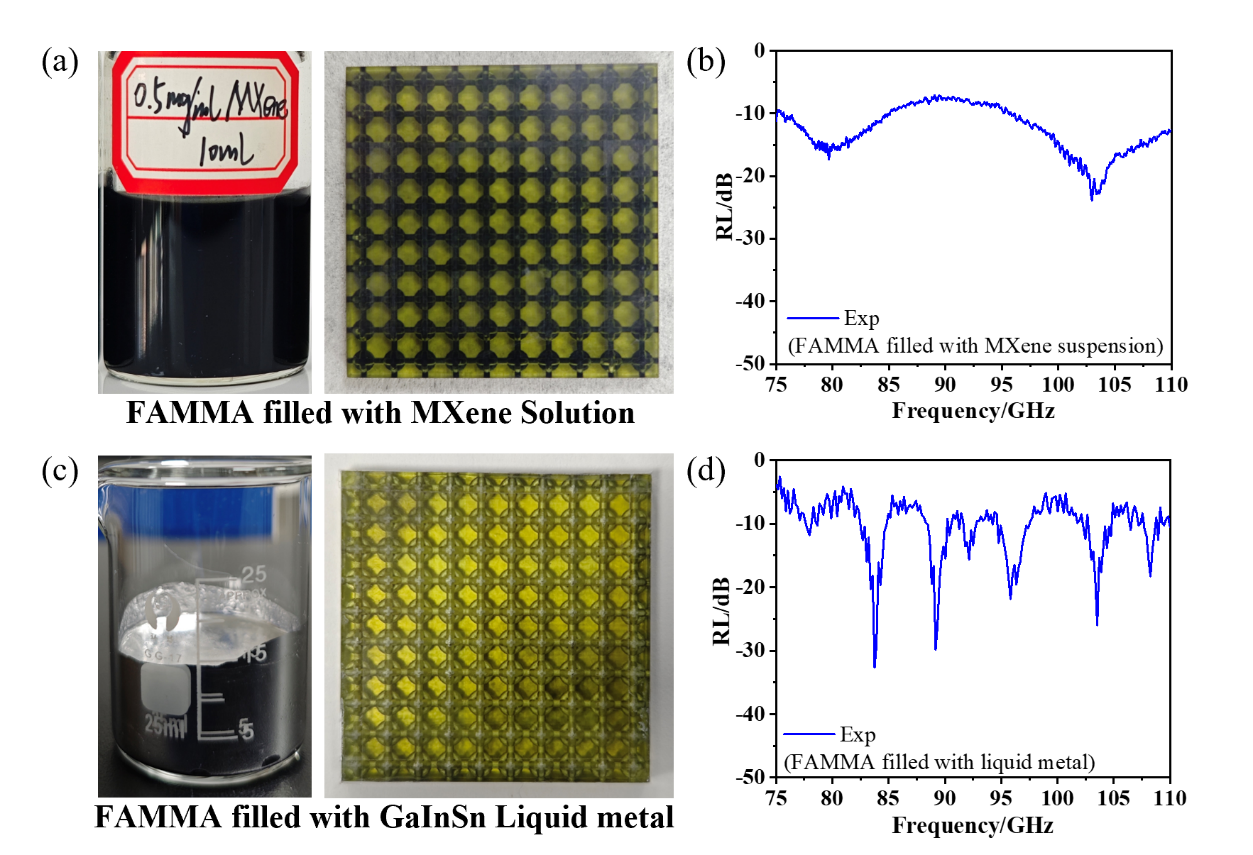
**

**Fig. S7** FAMMA with alternative dielectric medium. (**a**) Pictures of Mxene suspension and FAMMA filled with MXene suspension. (**b**) Measured RL of MXene suspension-filled FAMMA. (**c**) Pictures of GaInSn liquid metal and FAMMA filled with GaInSn liquid metal. (**d**) Measured RL of liquid metal-filled FAMMA


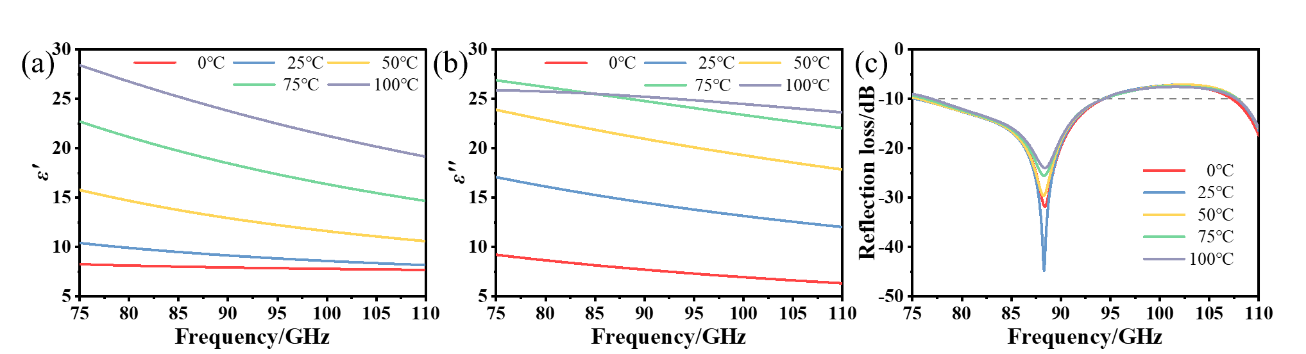


**Fig. S8** Variation of relative permittivity of water with frequency and temperature (**a**) real part of the relative permittivity; (**b**) imaginary part of the relative permittivity. (**c**) Simulation results of reflection loss of water-based FAMMA at different temperatures

“Regarding thermal stability, we have simulated the EMW absorption performance of water-based FAMMA at different temperatures using the Debye model [S3] to describe the dielectric properties and loss characteristics of water in CST by **Eq. (14)**:

$$\begin{aligned} \varepsilon\left( \omega,T \right)=\varepsilon_{\infty}\left( T \right)+\frac{\varepsilon_{s}\left( T \right)-\varepsilon_{\infty}\left( T \right)}{1-i\omega\tau\left( T \right)}\#\left( 14 \right) \end{aligned}$$

where ***ε_s_*** is the static dielectric constant, ***ε_∞_*** is the optical dielectric constant, *τ* is the rotational relaxation time, and *T* is the temperature of water. The temperature dependent parameters are defined via **Eqs. (15-17)**:

$$\begin{aligned} \varepsilon_{s}\left( T \right)=a_{1}-b_{1}T+c_{1}T^{2}-d_{1}T^{3}\#\left( 15 \right) \end{aligned}$$

$$\begin{aligned} \varepsilon_{\infty}\left( T \right)=\varepsilon_{s}\left( T \right)-a_{2}e^{-b_{2}T}\#\left( 16 \right) \end{aligned}$$

$$\begin{aligned} \tau\left( T \right)=c_{2}e^{\frac{T_{1}}{{T+T}_{0}}}\#\left( 17 \right) \end{aligned}$$

where $a_{1}$= 87.9, $b_{1}$= 0.404 K^-1^, $c_{1}$= 9.59×10^-4^ K^-2^, $d_{1}$= 1.33×10^-6^ K^-3^,

$a_{2}$= 80.7, $b_{2}$= 4.42×10^-3^ K^-1^, $c_{2}$= 1.37×10^-13^ s, $T_{0}$= 133°C, $T_{1}$= 651°C.

The results show the changes of complex permittivity and complex permeability of water with temperature. The absorption properties of water-based FAMMA at different temperatures can be obtained by importing the results into CST for simulation.”


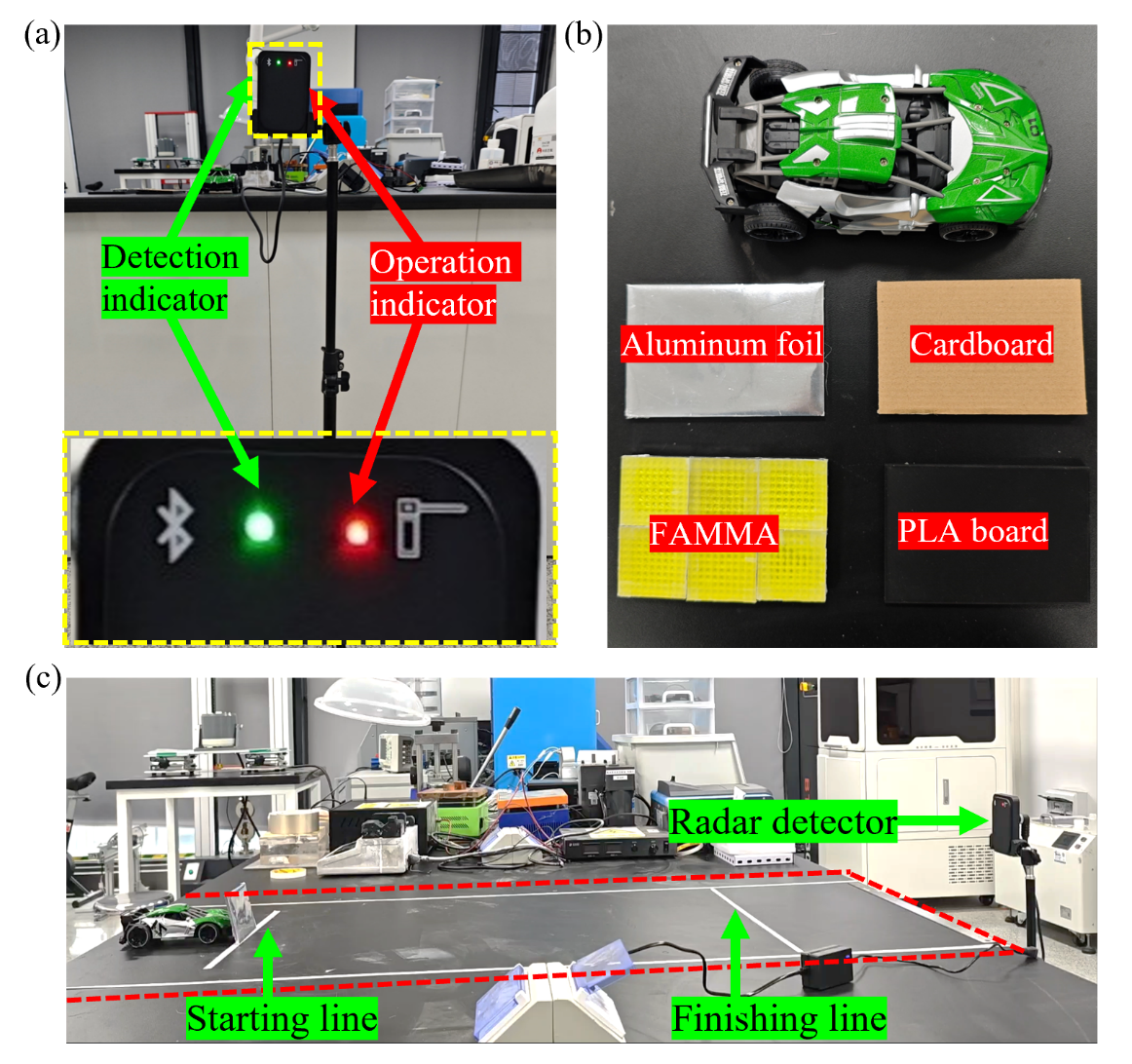


**Fig. S9** Radar detection applications. (**a**) Radar detection and working status schematic diagram; (**b**) The remote control car used in the experiment and four different tablets prepared. (**c**) Schematic diagram of radar detection application site

Four polylactic acid (PLA) plates with a geometric dimension of 12 mm×8 mm×5 mm were 3D printed using a fused deposition modeling (FDM) 3D printer (Bambu Lab X1) and used to shield the model car. A total of six FAMMA-based MMA, each comprising a 10 × 10 array of FAMMA, were used to shield the model car. Aluminum foil and honeycomb carboard shielding were cut to the size required to shield the model car.


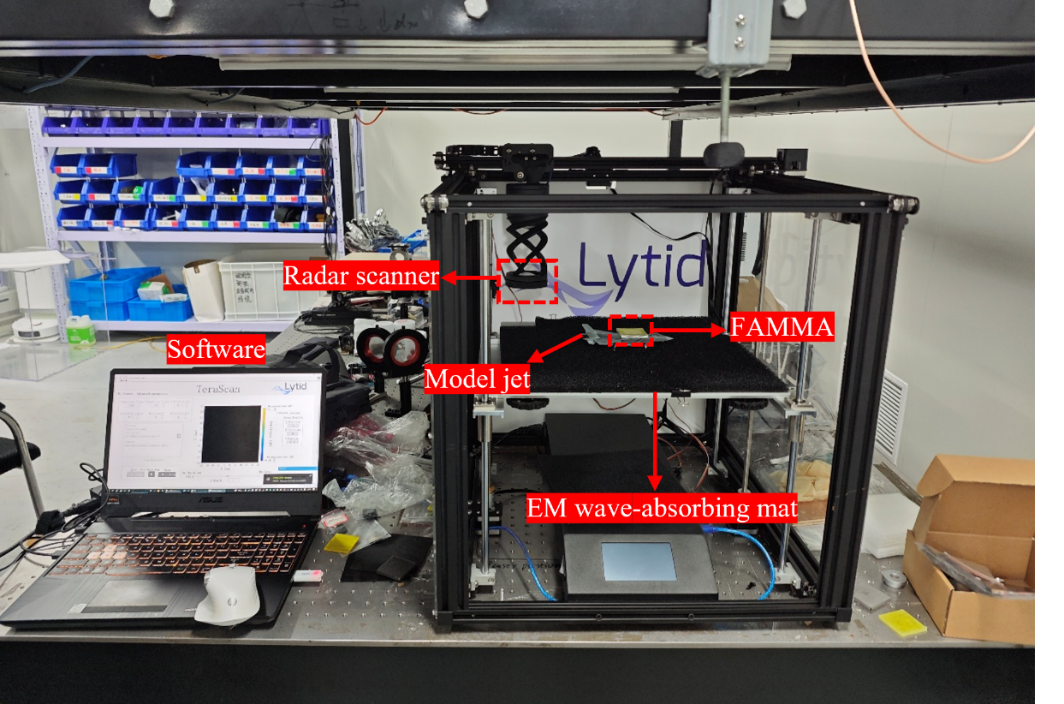


**Fig. S10** Experiment setup of FMCW radar imaging system

The specimen to be tested was placed in the middle of the platform. The radar scanner, controlled by a software, automatically scan the platform and generate an image of the specimen in real-time based on received signals.

**Supplementary References**

1. G. Deng, W. Chen, Z. Yu, F. Cai, J. Yang et al., 3D-printed dielectric-resonator-based ultra-broadband microwave absorber using water substrate. J. Electron. Mater. **51**(5), 2221–2227 (2022). https://doi.org/10.1007/s11664-022-09439-4
2. P. Tiwari, S.K. Pathak, in *2021 IEEE Indian Conference on Antennas and Propagation (InCAP)* (eds Priyanka Tiwari & Surya Kumar Pathak), pp. 692-695 (2021).
3. J. Wen, Q. Ren, R. Peng, Q. Zhao, Multi-functional tunable ultra-broadband water-based metasurface absorber with high reconfigurability. J. Phys. D Appl. Phys. **55**(28), 285103 (2022). <https://doi.org/10.1088/1361-6463/ac683e>
4. S. Li, Z. Shen, W. Yin, L. Zhang, X. Chen, 3D-printed terahertz metamaterial for electromagnetically induced reflection analogue. J. Phys. D Appl. Phys. **55**(32), 325003 (2022). <https://doi.org/10.1088/1361-6463/ac708c>
5. Z. Shen, S. Li, Y. Xu, W. Yin, L. Zhang et al., Three-dimensional printed ultrabroadband terahertz metamaterial absorbers. Phys. Rev. Applied **16**, 014066 (2021). <https://doi.org/10.1103/physrevapplied.16.014066>
6. W. Yin, S. Li, Z. Shen, Y. Cui, C. Yang et al., Broadband and multiband terahertz metamaterials based on 3-D-printed liquid metal-filled microchannel. IEEE Trans. Microwave Theory Techn. **71**(8), 3333–3340 (2023). <https://doi.org/10.1109/tmtt.2023.3278945>
